# Complete Genome Sequence of a Fowl Adenovirus D Strain Isolated from Chickens with Inclusion Body Hepatitis in Japan

**DOI:** 10.1128/MRA.00940-21

**Published:** 2021-11-18

**Authors:** Masaji Mase, Hiroshi Iseki, Satoko Watanabe

**Affiliations:** a National Institute of Animal Health, National Agriculture and Food Research Organization, Tsukuba, Ibaraki, Japan; b United Graduate School of Veterinary Sciences, Gifu University, Gifu, Gifu, Japan; c Graduate School of Life and Environmental Sciences, Osaka Prefecture University, Izumisano, Osaka, Japan; Queens College CUNY

## Abstract

We report the complete genome sequence of fowl adenovirus D (FAdV-D) strain JP/Tokushima/2010IBH, which was isolated from chickens with inclusion body hepatitis in Japan. This FAdV-D isolate was genetically highly similar to recent isolates from China, suggesting a common origin.

## ANNOUNCEMENT

Fowl adenovirus (FAdV) belongs to the family *Adenoviridae*, genus *Aviadenovirus* (1). FAdVs are classified into 12 serotypes (FAdV-1 to FAdV-8a and FAdV-8b to FAdV-11), which are grouped into five species (FAdV-A to FAdV-E) (1).

FAdV infections may be asymptomatic or may show symptoms associated with a variety of clinical and pathological conditions, including respiratory diseases, inclusion body hepatitis (IBH), enteritis, and other diseases found in chickens, quails, turkeys, pheasants, geese, and guinea fowls (1). Among them, IBH in chickens has been observed worldwide, and there are indications that its incidence is increasing in many types of poultry industries (2).

In Japan, IBH caused by FAdV-D has been frequently confirmed since 2010 (3). We determined and compared the complete genome sequence of a Japanese isolate with those from other countries to gain in-depth understanding of FAdV-D epidemiology.

The JP/Tokushima/2010IBH strain was used as an index strain for FAdV-D. This strain was isolated in 2010 from liver of IBH-affected chickens in Tokushima prefecture, located on Shikoku island, Japan (3). Primary chicken kidney cell (CKC) cultures prepared from 4- to 10-week-old chicks were used for the isolation and propagation of the viruses. Of the eight serotypes in Japan, JP/Tokushima/2010IBH was serologically neutralized only by SR48 (FAdV-2) antiserum (3, 4). For genetic analysis, the virus was propagated in CKC cultures and used after four passages, including two rounds of plaque purification. Viral DNA was then extracted from infected culture fluids using a QIAamp viral DNA microkit (Qiagen, Hilden, Germany). The genome was sequenced using Sanger sequencing, and the primer sequences used were based on a previous study (5). The inverted terminal repeat regions at the ends of the genome were determined using a modified rapid amplification of cDNA ends (RACE) technique.

The sequenced fragments were assembled using ATGC-Mac v.5 (Genetyx Corp., Tokyo, Japan). All tools were run with default parameters. The complete genome of JP/Tokushima/2010IBH was found to contain 43,833 nucleotides, with a GC content of 53.48%. The open reading frames were identified using Genetyx-Mac v.18 (Genetyx Corp.) and confirmed by alignment with published FAdV-D genomes. The gene order and shared open reading frames were also similar to those reported for FAdV-D strains such as HBQ12 and ON-P2 (5, 6).

Compared with FAdV sequences available in GenBank with BLASTN searches, JP/Tokushima/2010IBH showed 99.85 to 99.83% nucleotide sequence identity to the HBQ12 strain (GenBank accession number KM096545), which had been isolated from livers of chickens with IBH in China and genetically typed as FAdV-11 (5). The high percentage of genetic similarity between the two strains suggests that they were derived from the same origin.

The major difference between IBH-derived strains and non-IBH-derived strains is the number of 135-bp tandem repeat (TR) sequences (6, 7), with 3 to 7 TRs in IBH-derived strains and ≥10 TRs in non-IBH-derived strains (6). There were 5 TRs in HBQ12 and 4 TRs in JP/Tokushima/2010IBH.

**FIG 1:**
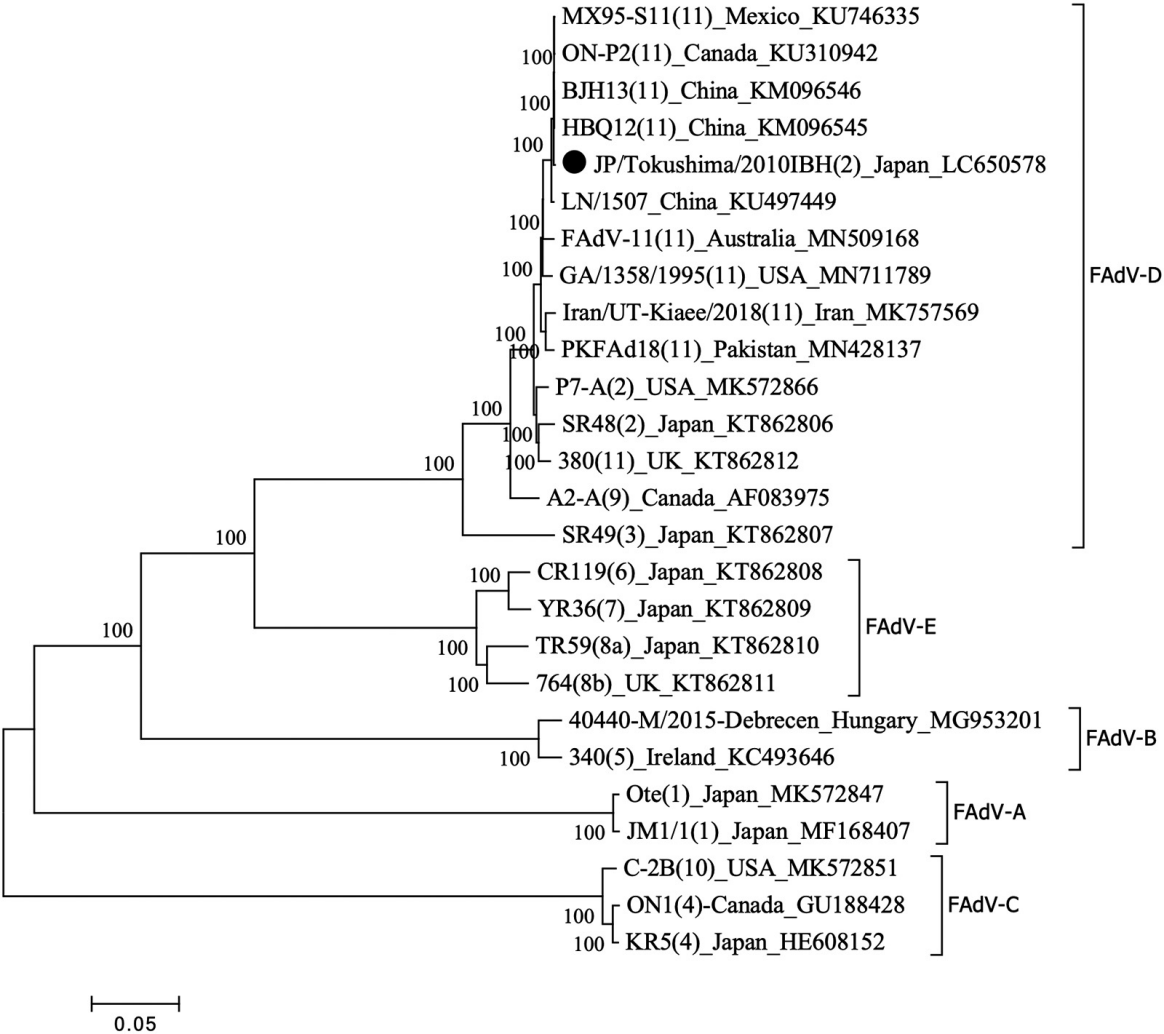
Phylogenetic tree based on the complete genome sequences of FAdV. The multiple alignment with available sequences from GenBank was conducted using the ClustalW program in MEGA 7 (10), and the tree was constructed by the neighbor-joining method with 1,000 bootstrap replications. All tools were run with default parameters unless otherwise specified. Horizontal distances are proportional to the minimum number of nucleotide differences required to join nodes and sequences. For strains with a serotype described by the isolate name, its number is shown in parentheses. The JP/Tokushima/2010IBH strain (GenBank accession number LC650578) is indicated with a black circle.

As shown in Fig. 1, strains of types 2 and 11 are genetically highly similar to each other and are clustered in the same group; it was previously proposed that strain SR48, which was the index strain of type 2, be merged into serotype 11 (8). Our findings strongly support these strains being unified as FAdV-2/-11, and this classification is already being used by other researchers (9).

### Data availability.

The genome sequence of FAdV-D strain JP/Tokushima/2010IBH was deposited in GenBank under the accession number LC650578.
